# Effects of GnRH agonists on the expression of developmental follicular anti-mullerian hormone in varying follicular stages in cyclic mice *in vivo*

**DOI:** 10.3892/mmr.2015.3993

**Published:** 2015-06-24

**Authors:** JILIANG HUANG, XIAOYAN WANG, ZHILING LI, RUOWU MA, WANFEN XIAO

**Affiliations:** Reproductive Center, The First Affiliated Hospital of Shantou University Medical College, Shantou University, Shantou, Guangdong 515041, P.R. China

**Keywords:** gonadotrophin-releasing hormone agonist, anti-mullerian hormone, ovarian follicle, granulosa cells

## Abstract

Gonadotrophin-releasing hormone (GnRH) agonists (GnRHa) have been widely used to induce a state of downregulation for *in vitro* fertilization, and its direct effects on the pituitary are well known. However, the effects of GnRHa on the expression of anti-mullerian hormone (AMH) by follicles in varying stages *in vivo* remain to be fully elucidated. In the present study 84 cyclic mice were randomly divided equally into four GnRHa groups and three cyclic mice were used as a control group. The expression levels of AMH in follicles of varying stages between days 0 and 7 following GnRHa administration were quantified using immunohistochemistry. The expression of AMH in follicles at various stages revealed dynamic changes during the process of downregulation. AMH in primary follicles initially increased and then decreased gradually. In small and large preantral follicles and in granulosa cells (GCs) surrounding the oocyte of small antral follicles, the expression of AMH began to increase on day 1, was attenuated on day 2, and then increased to a peak. The expression levels of AMH in the GCs surrounding the basement membrane, in contrast to the GCs surrounding the oocyte, were significantly lower and did not increase on day 1. In all stages of follicles, the expression of AMH declined gradually between the peak level and last day of downregulation. On day 7, the varying follicular stages all expressed lower levels of AMH than on day 0. This decrease was more prominent in the higher dose groups, compared with the lower dose groups. In conclusion, GnRHa was observed to induce time-dependent changes in the expression of AMH at varying follicular stages, which occurred in a dose-dependent manner.

## Introduction

Anti-mullerian hormone (AMH), also termed mullerian inhibiting substance, is a dimeric glycoprotein member of the transforming growth factor-β superfamily (1,2). AMH was first detected using a radioimmunoassay in mature bovine follicular fluid (3). In females, the expression of AMH is confined to the granulosa cells (GCs) of follicles in the ovary. Its expression has been detected in the GCs of follicles in several species, including mice (4), rats (5,6), sheep (7) and humans (8). The expression of AMH begins in the primary follicles and peaks in the preantral and small antral follicles. However, the levels of AMH gradually decline during the final stages of folliculogenesis, are not expressed in primordial follicles and disappear in atretic follicles (8,9).

A previous study observed that AMH can inhibit the initial recruitment of primordial follicles (10) and tumour cell proliferation (11). Other studies performed in animals have revealed that AMH can attenuate the sensitivity of follicles to follicle-stimulating hormone (FSH), reduce aromatase activity and the quantity of LH receptors in FSH-stimulated granulosa cells (12), and inhibit testosterone synthesis by thecal cells (13) by the binding of AMH to specific type II receptors (AMHRII) expressed in granulosa and theca cells. Previous detailed investigations have validated the use of serum AMH levels as a quantitative marker for ovarian reserve and ovarian dysfunction in *in vitro* fertilization (IVF) (14).

Gonadotrophin-releasing hormone (GnRH) agonist (GnRHa), a decapeptide, is similar in structure to native GnRH and binds to the GnRH receptors. Compared with native GnRH, GnRHa has a higher affinity for GnRH receptors of the pituitary, higher resistance to enzymatic breakdown and a prolonged half-life, leading to its slow dissociation from GnRH receptors (15) and the concomitant desensitization of pituitary GnRH receptors. This hypogonadotropic state is referred to as downregulation (15). In the 1980s, GnRHa was experimentally introduced in the mid-luteal phase of the preceding cycle during controlled ovarian hyperstimulation (COH) (16). By inhibiting the pituitary-ovary axis, GnRHa prevented premature luteinizing hormone (LH) surges during COH.

The way in which the levels of AMH in Uthe serum change during the process of downregulation remains controversial. A previous study found that AMH levels in the sera of patients with stage II-IV endometriosis are normal 4 and 8 weeks following prolonged downregulation with goserelin administration (17). However, another report found that circulating AMH declines following the first 3 months of prolonged GnRHa treatment in girls with central precocious puberty and early puberty (18). In accordance with a GnRHa-mediated decrease in serum levels of AMH, a reduction in the levels of AMH were observed following GnRHa treatment in premenopausal women with breast cancer (19). It has been suggested that GnRHa increases the mRNA expression of AMH in cultured human granulosa cells (hGCs) and in the human HGL5 granulosa cell line (20). By contrast, evidence implying a direct effect of GnRHa on suppression of AMH expression in the ovaries has been obtained from observations that AMH concentrations in follicular fluid from females treated with GnRHa are lower, compared with untreated controls (21).

Previous studies on the effects of GnRHa on the expression of AMH have all involved examination of the serum levels of AMH *in vivo* and GCs *in vitro*. The effects of GnRHa *in vivo* on the expression of AMH in follicles at varying follicular stages during downregulation remain to be elucidated. Understanding the changes of AMH in GCs can enable verification of serum AMH and provide evidence supporting the further utilization of serum AMH. The purpose of the present study was to investigate the effects of different doses of GnRHa administration on the expression of AMH at varying follicular stages during the process of downregulation *in vivo*.

## Materials and methods

### Animals

All experimental procedures in the present stduy were performed at the Experimental Animal Centre of Shantou University Medical College (SUMC; Shantou, China), according to the international ethical guidelines and with the approval of the SUMC Ethics Committee. (SUMC2013-129). Adult female KM mice (5-6 weeks old) were obtained from the Animal Center of SUMC and were housed under a 12/12 h light/dark cycle at 22-26°C and 50-60% humidity. All mice had free access to a standard pellet diet and water. The estrous cycles of the adult female mice were determined in vaginal smears, which were collected for 21 consecutive days. Only mice that had more than two consecutive regular 4-day cycles were used. These mice were randomly allocated into four experimental groups, with 21 mice per group, as well as a control group containing three mice.

### Treatment

Treatment for downregulation involved a daily intraperitoneal injection of GnRH agonist (Triptorelin; Ipsen Pharma Biotech, France) between days 3 and 9 of the estrus cycle at the following doses: 1.5 *µ*g/100 g body weight (bw)/day, 3.0 *µ*g/100 g bw/day, 4.5 *µ*g/100 g bw/day and (4) 6.0 *µ*g/100 gbw/day. Control mice remained without any treatment.

### Tissue collection

In each treatment group, three mice were sacrificed each day following GnRH agonist administration, up to day 9. The mice in the control group were sacrificed on day 0, corresponding to day 3 of the estrus cycle. The ovaries were removed from each animal and cleaned of surrounding fat.

### Immunohistochemistry

The ovaries were fixed in 4% para-formaldehyde at 4°C overnight and embedded in paraffin. The ovaries were then serially sectioned at a thickness of 4 *µ*m. Every 10th section was used. The tissue sections between days 0 and 7 were obtained at the same time for each trial. Following dewaxing in xylene, rehydration in a series of ethanols and antigen retrieval in a microwave oven (15 min), endogenous peroxidase activity was blocked with 3% H_2_O_2_ for 15 min. To block non-specific binding sites, the sections were then incubated in 10% normal donkey serum diluted in 0.01 M phosphate-buffered saline (PBS) for 15 min. The sections were incubated at 4°C overnight with polyclonal goat anti-MIS antibody (cat. no. sc-6886, Santa Cruz Biotechnology, Santa Cruz, CA, USA), diluted 1:50 in 0.01 M PBS. The sections were then incubated for 1 h with secondary horseradish peroxidase (HRP)-conjugated donkey anti-goat IgG-HRP (cat. no sc-2020; Santa Cruz Biotechnology), diluted 1:100 in 0.01 M PBS. The detection of AMH was performed using chromogen, 3.3-diaminobenzidine (DAB; Gene Tech Shanghai Company Limited), according to the manufacturer's instructions. The sections were counterstained with hema-toxylin, and were then dehydrated and mounted. For a negative control, the primary antibody was excluded.

### Quantification of immunohistochemistry

The morphological classification of different stages of follicles were, as defined previously (22,23). The follicles were distinguished as follows: Primordial follicles exhibited a single layer of flattened GCs; primary follicles exhibited a single layer of cuboidal GCs; small preantral follicles possessed between two and five layers of GCs; large preantral follicles possessed more than five layers of GCs without an antrum; small antral follicles contained fluid-filled spaces with fewer than five GC layers, and follicles with five or more layers of GCs and an antrum were considered large antral follicles. Only follicles that contained a visible oocyte with a nucleus were selected for immunohistochemical quantification.

### Expression levels of AMH in follicles at different developmental stages, at different administration times and with different doses of GnRHa

For comparison of the expression levels of AMH in the control and treatment groups between days 1 and 7 (seven subgroups each day, n=3 per subgroup), 20 follicles of each stage in each animal were examined. For the control group (day 0; n=3), 20 follicles of each stage in each animal were also examined. To quantify the mean density of the expression of AMH from individual follicles, analysis was performed using an image analysis system linked to an Olympus camera (Olympus Corporation, Tokyo, Japan). All images were captured under the same exposure times, and the mean density of each follicle was measured using Image-Pro Plus 6.0 (Media Cybernetics, Inc., Rockville, MD, USA)

### Expression of AMH in the GCs surrounding the oocyte and basement membrane of small antral follicles with different doses of GnRHa

Additional analysis was performed to determine the mean density from different compartments of AMH staining within each follicle in the small antral follicles. The area of the GCs surrounding the oocyte and basement membrane were independently calculated using the same image analysis technique described above for the different follicular stages.

### Statistical analysis

All data are expressed as the mean ± standard error of the mean. Statistical analysis was performed and differences between the respective treatments performed and the control were determined using one-way analysis of variance followed by Dunnett's multiple comparison test. P<0.05 was considered to indicate a statistically significant difference.

## Results

### Pattern and location of the expression of AMH during follicular development

**Figure 1:**
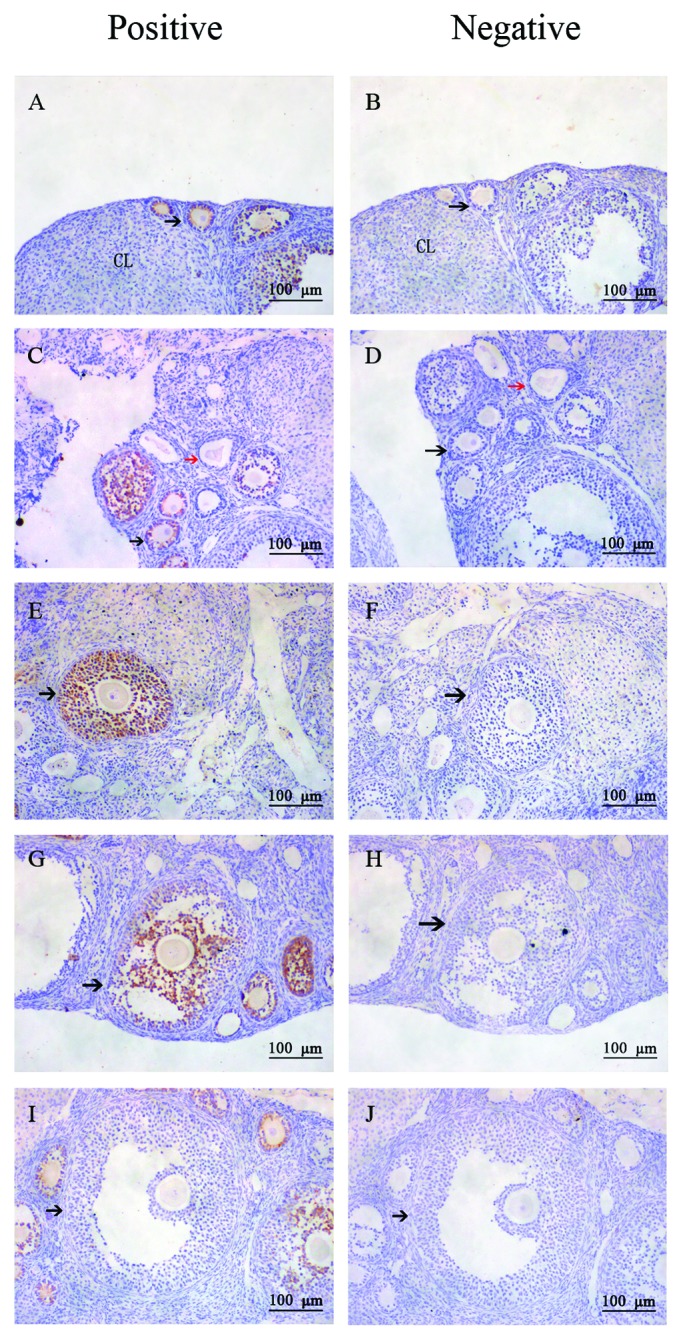
Immunohistochemical localization of the expression of AMH in different follicular stages. Histological ovarian sections (magnification, x200). Positive immunohistochemical staining of AMH in follicles at varying stages are shown on the left; negative control stains are shown on the right. Primary (A and B, black arrow), small preantral (C and D, black arrow), large preantral (E and F, black arrow), small antral (G and H, black arrow), and large antral (I and J, black arrow) follicles, corpus luteum (A and B, CL), atretic follicles (C and D, red arrow). Scale bar=100 *µ*m. AMH, anti-mullerian hormone.

The results of the immunohistochemistry revealed that the expression of AMH was positive in The GCs from the primary follicles and was confined to the cytoplasm (Fig. 1). The expression of AMH increased with decreasing size of the follicles. The AMH immunostaining was most marked in the preantral and small antral follicles, and was absent in the large antral follicles. In the atretic follicles and primordial follicles, no AMH was detected. At the early antral stage, AMH was predominantly present in the GCs surrounding the oocyte and in a few cells surrounding the antrum. No follicles expressed AMH in the thecal layer. Only GCs of the primary follicles exhibited homogeneous expression of AMH. Similar to the physical status, GnRHa treatment did not change the pattern of stage-specific expression of AMH or the location of expression in any of the four treatment groups. No specific immunoreactivity was observed in the negative-control ovaries.

### Effects of GnRHa on the expression levels of AMH in primary follicles

**Figure 2:**
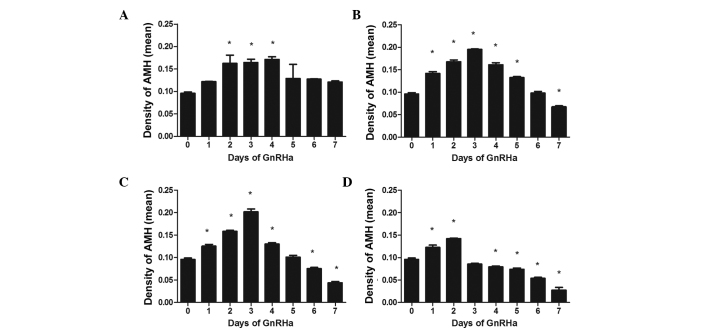
Time-dependent effects of GnRHa on the expression of AMH in primary follicles. The average AMH levels at (A) 1.5 *µ*g/100 g, (B) 3.0 *µ*g/100 g, (C) 4.5 *µ*g/100 g and (D) 6.0 *µ*g/100 g GnRHa are presented. Values are expressed as the mean ± standard error of the mean. ^*^P<0.05, compared with day 0. AMH, anti-mullerian hormone; GnRHa, gonadotrophin-releasing hormone agonist.

Fig. 2 shows the effect of treatment with different doses of GnRHa on the expression of AMH in primary follicles. The expression of AMH expression increased initially, peaking at ~day 3, and then declined gradually. This peak moved forward from day 4 to day 2 as the dose of GnRHa increased, with the peak AMH values all significantly different to those on day 0 (all P<0.05). AMH decreased gradually following the peak, with no significant difference at the lowest dose (Fig. 2A). However, significant differences were observed at the highest three doses on day 7 (Fig. 2B–D).

### Effects of GnRHa on the expression of AMH in small preantral follicles

**Figure 3:**
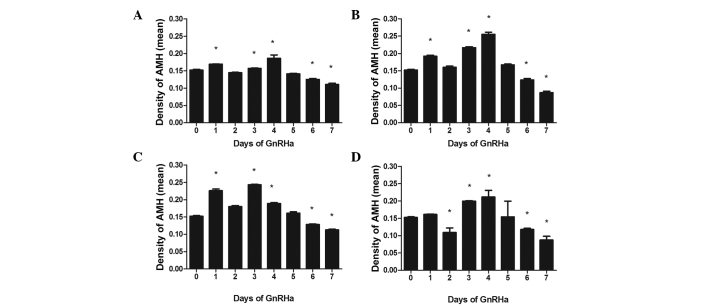
Time-dependent effects of GnRHa on the expression of AMH in small preantral follicles. The levels of AMH at (A) 1.5 *µ*g/100 g, (B) 3.0 *µ*g/100 g, (C) 4.5 *µ*g/100 g and (D) 6.0 *µ*g/100 g GnRHa are presented. Values are expressed as the mean ± standard error of the mean. ^*^P<0.05, compared with day 0. AMH, anti-mullerian hormone; GnRHa, gonadotrophin-releasing hormone agonist.

In the three lower dose treatment groups, the expression of AMH increased on days 1 and 2 to a significant degree (all P<0.05; Fig. 3). However, the highest dose treatment group caused no elevation in the level of AMH on day 1 (P>0.05), and were observed to decline on day 2, with levels significantly lower than those observed on day 0 (P<0.05). Following the decrease on day 2, the levels of AMH increased, and peaked on day 4. With continuous administration of GnRHa, the expression of AMH decreased to a significantly lower level on day 7, subsequent to the peak in all four treatment groups (P<0.01).

### Effects of GnRHa on the expression of AMH in large preantral follicles

**Figure 4:**
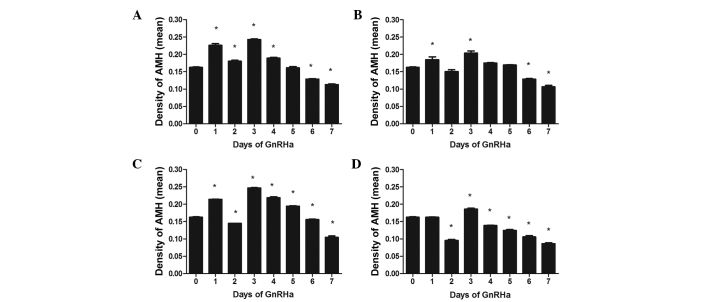
Time-dependent effects of GnRHa on the expression of AMH in large preantral follicles. The average AMH levels at (A) 1.5 *µ*g/100 g, (B) 3.0 *µ*g/100 g, (C) 4.5 *µ*g/100 g and (D) 6.0 *µ*g/100 g GnRHa are presented. Values are expressed as the mean ± standard error of the mean. ^*^P<0.05, compared with day 0. AMH, anti-mullerian hormone; GnRHa, gonadotrophin-releasing hormone agonist.

Similar to the observations in the small preantral follicles, the intensity of AMH staining was increased on day 1 in the large preantral follicles, with a significant difference in the three lower dose groups (P<0.01), but no significant difference in the highest dose group (P>0.05). On day 2, the expression of AMH in all the groups reduced, compared with those on day 1, although the level of AMH in the lowest dose group remained significantly higher than that at day 0 (P<0.01; Fig. 4A). The levels of AMH in the remaining three groups were all lower than those on day 0, with differences becoming increasingly significant with increasing GnRHa dose. All peaks in AMH occurred on day 3. Following this peak, the levels of AMH levels declined gradually, with all groups exhibiting significantly lower levels by day 7, compared with day 0 (all P<0.01).

### Effects of GnRHa on the expression of AMH expression in small antral follicles

**Figure 5:**
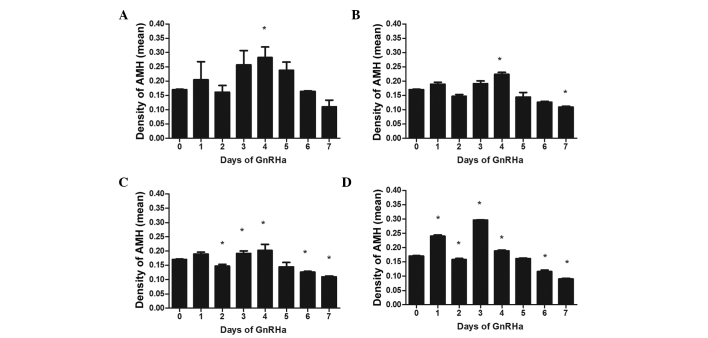
Time-dependent effects of GnRHa dose on AMH expression in small antral follicles. The average AMH levels at (A) 1.5 *µ*g/100 g, (B) 3.0 *µ*g/100 g, (C) 4.5 *µ*g/100 g and (D) 6.0 *µ*g/100 g GnRHa are presented. Values are expressed as the mean ± standard error of the mean. ^*^P<0.05, compared with day 0. AMH, anti-mullerian hormone; GnRHa, gonadotrophin-releasing hormone agonist.

The AMH staining observed in the GCs of the small antral follicles increased in all GnRHa treatment groups on day 1, however, only the increase in the highest GnRHa treatment group was statistically significant to that on day 0 (P<0.01; Fig. 5). All treatment groups exhibited reduced expression levels of AMH on day 2, compared with day 0, with the difference being significant in the two highest dose groups (P<0.05). The peak in expression occurred on day 4 in the three lower dose groups, and on day 3 in the highest dose group. The expression levels of AMH declined gradually following the peak in all treatment groups. The decrease on day 7 was statistically significant, compared with day 0, in the three highest dose groups (P<0.05), while no difference was observed in the lowest dose group (P>0.05).

### Effects of GnRHa on the expression of AMH in the GCs surrounding the oocyte and basement membrane of the small antral follicles

**Figure 6:**
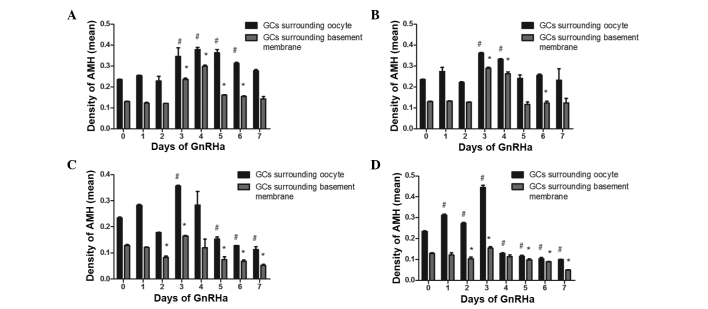
Time-dependent effects of GnRHa dose on the expression of AMH in the GCs surrounding the oocyte and basement membrane of small antral follicles. The average AMH levels at (A) 1.5 *µ*g/100 g, (B) 3.0 *µ*g/100 g, (C) 4.5 *µ*g/100 g and (D) 6.0 *µ*g/100 g GnRHa are presented. The black columns indicate the mean value of AMH in the GCs surrounding the oocyte. The grey columns indicate the mean value of AMH in the GCs surrounding the basement membrane. Values are expressed as the mean ± standard error of the mean. ^*^P<0.05, compared with day 0. AMH, anti-mullerian hormone; GnRHa, gonadotrophin-releasing hormone agonist; GCs, granulosa cells.

Fig. 6 shows the changes in the expression of AMH in the GCs surrounding the oocyte and basement membrane. In the GCs surrounding the oocyte, a significant increase in the expression of AMH was observed on day 1 in the highest dose group only (P<0.01; Fig. 6D). On day 2, the intensity of AMH staining decreased in all groups, compared with day 1, however, the expression of AMH in the highest dose group was significantly higher than that on day 0 (P<0.01). Peak levels occurred on day 3 in all groups but the lowest dose group, which peaked on day 4, following which the levels of AMH slowly declined. The levels of AMH in the two lowest dose groups returned to basal levels on day 7 (P>0.05). However, a rapid decrease resulting in a significantly lower level was observed on day 7, compared with day 0, in the two highest dose (all P<0.01).

Regarding the GCs surrounding the basement membrane, no significant changes in AMH were observed in any group on day 1. However, the two highest dose groups exhibited significant decreases in the expression levels of AMH on day 2 (P<0.05). The AMH peaks in the GCs surrounding the basement membrane were synchronous with those of the GCs surrounding the oocyte in all groups (P<0.05). In all groups, the expression of AMH declined following this peak. In the two lowest dose groups, no significant difference was observed in the AMH levels on day 7, compared with day 0 (P>0.05). In the two highest dose groups, significantly lower levels were observed on day 7, compared with those on day 0 (P<0.05).

## Discussion

Serum AMH is affected by the expression of AMH in various follicular stages, however, the way in which AMH levels in the serum change during the process of downregulation remains to be fully elucidated, and reports on whether the expression of AMH in the GCs of follicles at different growing stages is affected by GnRHa *in vivo* are lacking. In the present study, it was demonstrated for the first time, to the best of our knowledge, that the expression of AMH in follicles at various stages change dynamically following treatment with various doses of GnRHa *in vivo*, during the process of downregulation.

In the mouse ovary, the present study demonstrated that the expression of AMH in follicular GCs was stage-specific. AMH protein was produced at low levels by the columnar GCs of the primary follicles, and was most abundant in the GCs of the preantral and small antral follicles. The expression of AMH was terminated in the large antral follicles and the corpora lutea, and was absent in the primordial and atretic follicles. No AMH was observed in the thecal or interstitial cells. These results are consistent with previous findings (4,24). In the present study, treatment with GnRHa *in vivo* dids not affect the stage-specific location of the expression of AMH. In addition, it was also observed that the intensity of the expression of AMH was homogeneous in the primary follicle GCs, but heterogeneous in the preantral and small antral follicles. In primary follicles, apoptosis seldom occurs in human ovarian tissue sections (25). Similar homogeneity in the expression of AMH in all the GnRHa groups in the present study is indirectly supported by investigations performed in hypophysectomized, estrogen-treated immature rats, which suggested that GnRHa does not induce apoptosis in the GCs of primary follicles (26). However, in follicles between the small preantral and small antral stages, physical apoptosis, along with the proliferation of granulosa cells and GnRHa induce pharmacological apoptosis by binding GnRH receptors.

In the present study, the expression of AMH expressions exhibited a similar dynamic change in all follicular stages all treatment groups, with the exception of the decrease observed on day 2 from the small preantral stages onward. The levels of AMH were observed to initally increase, and then gradually decrease. The peak of AMH occured on ~day 3. GnRHa decreased the levels of AMH below their initial levels by day 7, which occurred in a dose-dependent manner. Furthermore, from the peak onwards, the expression of AMH declined in a time-dependent manner. In general, the AMH profile in the GCs of the small antral follicles was similar to that of preantral the follicles. It is evident that antrum formation physically demarcates the former preantral GCs into mural and cumulus GC populations (27). Notably, the present study demonstrated that the time-dependent dynamic changes in the expression of AMH in the GCs from the two different regions were different. Firstly, the AMH profile in GCs surrounding the oocyte was similar to that of the preantral follicles, while a marked difference was observed in the GCs surrounding the basement membrane, particularly on the first day of GnRHa administration, without a visible increase. This result confirmed that of a previous finding that cumulus cells are more closer associated with preantral GCs, compared with mural granulosa cells (28). Secondly, the expression of AMH in the small antral follicles was more abundant in the GCs surrounding the oocyte than in the GCs within the basement membrane, which was consistent with previous findings (5,24). The difference in AMH intensity indirectly demonstrated functional differences between the GCs in different locations. Follicle-stimulating hormone receptor (FSHR) is highly expressed in cumulus cells. By binding FSHR, FSH can induce the expansion of cumulus cells and is involved in oocyte maturation (29). In addition, it has been reported that the expression levels of FSHR and AMHR2 in cumulus cells is associated with the expression of AMH (30). Therefore, the response to FSH is more marked in GCs surrounding the oocyte than in the basement membrane, which partly explains their more visible ascent on day 1 in all groups surrounding the oocyte.

During the first few days of the downregulation, daily injection GnRHa causes an initial flare-up of gonadotropin concentration by acting at the level of the hypothalamo-hypophysis axis. Following this increase, the pituitary gland is downregulated to a hypogonadotropic state (31). Therefore, under the effects of GnRHa, gonadotropins first increase and then slowly decrease. Previous studies have demonstrated this dynamic change in FSH, LH and E2, and observed an increasing tendency on day 3 when GnRHa was administrated to rats (32). In the present study, a similar increasing tendency of AMH around day 3 was observed. Accordingly, the present study hypothesized that the expression of AMH is, at least partially, gonadotropin-dependent. This hypothesis is supported further by previous *in vitro* findings demonstrating the stimulation of AMH production in GCs following the addition of FSH (33). Similarly, experiments performed in primates also support a possible positive role for gonadotropins in the regulation of AMH (34). By contrast, other studies have revealed that FSH may downregulate the expression of AMH (19,24). Therefore, controversies exist regarding the effect of gonadotropins on the expression of AMH, although early investigations have reported that the expression of AMH is gonadotropin-independent (35,36).

Indirectly, the results of the present study suggested that the production of AMH in primary follicles is partially gonadotropin-responsive. Supporting this hypothesis are previous findings that FSH and LH receptor mRNAs exist in primary follicles (37,38). The time-dependent changes observed in the expression of AMH in preantral and small antral follicles were not entirely in accordance with the trend of gonadotropins, particularly on day 2, therefore, the production of AMH was not only positively regulated by gonadotropins, but also negatively regulated by other factors.

GnRHa acts predominantly on the hypothalamus-pituitary-gonad axis by inducing a hypogonadotropic milieu from continuous exposure to the agent. Apart from its pituitary actions, several studies have demonstrated that GnRHa can exert effects on the ovary in an autocrine-paracrine manner, as an intra-ovarian regulatory factor, particularly by affecting follicular development and steroidogenesis (39,40). This direct GnRHa effect on the ovary is supported by increasing evidence that GnRH receptors exist in ovarian tissue (39,41-46). One of the direct actions on the ovary is inhibition of the expression of gonadotropin receptors in granulosa cells (47). These findings suggest that the anti-gonadotropic effect by GnRHa contributes to the reduction of AMH. The results of a previous study revealed that lower levels of AMH in intrafollicular fluid are found in females treated with GnRHa (21). According to previous studies, GnRHa directly inhibits proliferative activity and induces apoptosis in GCs from preantral and small antral follicles (21,26,48), and the incidence of apoptosis increases in a dose-dependent manner (49). Other studies have reported that the expression of AMH is associated with the proliferation of GCs (5). The present study hypothesized that the downregulation of the expression of AMH by GnRHa results from an inhibitory effect of mitotic activity and induction of apoptosis, although the mechanisms remain to be elucidated.

In the present study, the protein expression pattern of AMH in primary follicles on day 2 was different from that of the preantral and small antral follicles. The discrepancy in the density of GnRH receptors may, at least in part, explain this difference, GnRH receptors have been reported to localize in granulosa cells of the preantral and small antral follicles, whereas no GnRH receptors exist in primary follicles (45). The present study hypothesized that the positive effects of FSH on the expression of AMH expression were partially counteracted by the negative effects of GnRHa through acting on GnRH receptors, leading to a significant decrease in the levels of AMH on day 2 in the preantral and antral follicles. In preovulatory rat GCs, GnRH induces an increase in the receptor levels in a dose-dependent manner (50). This may partly explain the finding that the expression of AMH in the preantral and small antral follicles was lowest at the end of downregulation in the group treated with the highest dose of GnRHa. As for the degree of decrease in the expression of AMH in the GCs surrounding the oocyte and basement membrane of the small antral follicles, GnRH receptors may explain this discrepancy. An increased density of GnRH receptors exist in the cumulus cells surrounding the oocyte, compared with mural GCs (45). Therefore, inhibition of AMH by GnRHa may be higher in GCs surrounding the oocyte.

During the process of downregulation to induce a hypogonadotropic state, GnRHa causes an initial 'flare-up' effect, resulting in a transitory increase of gonadotrophins. This effect accelerates follicle recruitment (51). In addition, it is now evident that AMH is involved in mouse primordial follicle selection (9,52), and growing follicle cyclic recruitment in humans (8) and mice (53). AMH inhibits FSH-stimulated follicle growth by decreasing the responsiveness of the follicle to FSH (53,54). The inhibitory effect of AMH on the expression of aromatase activity and LH receptors by cultured GCs is also in agreement with the inhibitory effect on follicle growth (55). Therefore, it was suggested that the decrease of AMH observed in the present study on day 2 accelerated follicle recruitment, while the increase of AMH around the peak day restricted further follicular development in an autocrine-paracrine manner. Following the the 'flare-up,' FSH decreases gradually. In the hypogonadotropic state, follicle development is relatively static. The low expression levels of AMH on day 7 may have been associated with increased sensitivity to FSH, allowing follicles to be selected synchronously for continued growth by exogenous application of gonadotrophins. This suggested that AMH may be one of the factors involved in follicular synchronicity.

Follicular development is a process not only regulated by the hypothalamic-pituitary-ovarian axis, but is also affected by autocrine/paracrine factors of the ovaries (51). A bidirectional communication between oocytes and GCs contributes to follicular development (56). The oocyte itself has effects on gene expression and protein synthesis in the GCs by secreting paracrine factors, and GCs regulate oocyte developmental competence (57). Treatment of GnRHa *in vivo* may affect the communication between the oocyte and GCs, by either changing the function of the GCs or modulating the response of GCs to paracrine factors secreted by the oocyte, leading to changes in the expression of AMH. The expression and secretion of AMH by cumulus GCs is correlated with oocyte maturity, with an inverse association between the expression of AMH and oocyte maturity (58). This suppression of oocyte maturation by AMH has been demonstrated previously (59). In addition, observations from the rat model also support this inhibitory role of AMH (60). Therefore, the downregulation of AMH observed on day 7 in the presents study may be a marker of oocyte quality, and it was hypothesized that appropriate treatment with GnRHa *in vivo* may improve oocyte quality. Consistent with this hypothesis, a previous study performed in the mice demonstrated that administrating GnRH in addition to the standard pregnant mare serum gonadotropin (PMSG) and human chorionic gonadotropin (hCG) treatments improves IVF fertility rate, compared with standard treatments (61). This further supports the possibility that GnRHa *in vivo* may regulate follicle development and improve oocyte quality through changes in the expression of AMH during the process of downregulation.

The present study demonstrated that treatment of GnRHa *in vivo* affects the expression of AMH in follicles at different stages in the ovaries of cycling mice during the process of downregulation, however it has no affect on its expression pattern. The effect of GnRHa on the expression of AMH was dose-dependent. The dynamic changes observed in the expression of AMH in the present study may contribute to the use of GnRHa in regulating follicle development and improving oocyte quality during downregulation in assisted reproductive technology. However, the potential mechanisms underlying the daily fuctuations in the expression of AMH caused by GnRHa *in vivo* requires further investigation.

## Abbreviations

AMH: anti-mullerian hormone
AMHRII: anti-mullerian hormone type II receptor
ANOVA: analysis of variance
ART: assisted reproductive technology
COH: controlled ovarian hyperstimulation
FSH: follicle stimulating hormone
FSHR: FSH receptor
GCs: granulosa cells
GnRHa: gonadotrophin-releasing hormone agonist
hGCs: human granulosa cells
HGL5: human granulosa cell line
IVF: in vitro fertilization
LH: luteinizing hormone
MIS: mullerian inhibiting substance
PBS: phosphate buffer saline
PMSG: pregnant mare serum gonadotropin
